# Monoclonal antibodies indicate low-abundance links between heteroxylan and other glycans of plant cell walls

**DOI:** 10.1007/s00425-015-2375-4

**Published:** 2015-07-25

**Authors:** Valérie Cornuault, Fanny Buffetto, Maja G. Rydahl, Susan E. Marcus, Thomas A. Torode, Jie Xue, Marie-Jeanne Crépeau, Nuno Faria-Blanc, William G. T. Willats, Paul Dupree, Marie-Christine Ralet, J. Paul Knox

**Affiliations:** Centre for Plant Sciences, Faculty of Biological Sciences, University of Leeds, Leeds, LS2 9JT UK; UR1268 Biopolymères, Interactions et Assemblages, Institut National de la Recherche Agronomique, Rue de la Géraudière, BP 71627, 44316 Nantes, France; Department of Plant and Environmental Sciences, University of Copenhagen, Thorvaldsensvej 40, 1871 Frederiksberg C, Denmark; Department of Biochemistry, University of Cambridge, Tennis Court Road, Cambridge, CB2 1QW UK

**Keywords:** Arabinogalactan-proteins, Cell wall, Glucuronoxylan, Pectin, Polysaccharides, Rhamnogalacturonan-I

## Abstract

**The derivation of two sensitive monoclonal antibodies directed to heteroxylan cell wall polysaccharide preparations has allowed the identification of potential inter-linkages between xylan and pectin in potato tuber cell walls and also between xylan and arabinogalactan-proteins in oat grain cell walls.**

Plant cell walls are complex composites of structurally distinct glycans that are poorly understood in terms of both *in muro* inter-linkages and developmental functions. Monoclonal antibodies (MAbs) are versatile tools that can detect cell wall glycans with high sensitivity through the specific recognition of oligosaccharide structures. The isolation of two novel MAbs, LM27 and LM28, directed to heteroxylan, subsequent to immunisation with a potato cell wall fraction enriched in rhamnogalacturonan-I (RG-I) oligosaccharides, is described. LM27 binds strongly to heteroxylan preparations from grass cell walls and LM28 binds to a glucuronosyl-containing epitope widely present in heteroxylans. Evidence is presented suggesting that in potato tuber cell walls, some glucuronoxylan may be linked to pectic macromolecules. Evidence is also presented that suggests in oat spelt xylan both the LM27 and LM28 epitopes are linked to arabinogalactan-proteins as tracked by the LM2 arabinogalactan-protein epitope. This work extends knowledge of the potential occurrence of inter-glycan links within plant cell walls and describes molecular tools for the further analysis of such links.

**Electronic supplementary material:**

The online version of this article (doi:10.1007/s00425-015-2375-4) contains supplementary material, which is available to authorised users.

## Introduction

Plant cell walls are highly complex composites of macromolecules that are constructed from of a range of structurally varied polysaccharides/glycoconjugates that potentially include cellulose, pectins, xyloglucans, heteroxylans, heteromannans, mixed-linkage glucans and arabinogalactan-protein proteoglycans (AGPs). The biochemistry of these components has been studied extensively, and in broad terms, most of the abundant and widespread structures are well characterised (Burton et al. 2010). However, the overall architectures of plant cell walls, the ways in which these components are organised, and variations in relation to cell types and developmental events largely remain unknown.

A range of models outlining the organisation of cell wall polymers has been presented (Keegstra et al. 1973; McCann and Roberts 1994; Carpita and Gibeaut 1993; Somerville et al. 2004; Baba 2006). Many of these depict cellulose microfibrils interlinked by xyloglucans/xylans and this scaffolding embedded in a pectic matrix. Recent work has allowed the development of a more nuanced view of cell wall matrix glycans including xyloglucans, heteroxylans and pectic supramolecules as all being components of cell wall matrices, and several of which may interact with cellulose microfibrils and several of which can be interconnected (Zykwinska et al. 2007; Burton et al. 2010; Cosgrove 2014). Proposed links between xyloglucan and pectins (Cumming et al. 2005; Popper and Fry 2008), the identification in Arabidopsis cell cultures of a low-abundance polymer, Arabinoxylan Pectin Arabinogalactan Protein1 (APAP1), consisting of AGP, rhamnogalacturonan-I (RG-I) and arabinoxylan domains (Tan et al. 2013) and evidence for enzyme capabilities leading to matrix glycan interlinks (Franková and Fry 2013) all support the idea of highly dynamic sets of matrix glycans in cell walls that can vary in configurations, inter-linkages, and properties. Pectic supramolecules appear to be a highly complex and heterogeneous components of cell wall matrices. The major polysaccharide domains of pectic molecules are homogalacturonan (HG) and the branched heterodomain rhamnogalacturonan-I (RG-I) containing side chains rich in galactan and arabinan motifs with other domains including rhamnogalacturonan-II and xylogalacturonan (Atmodjo et al. 2013). How these domains of supramolecules are ordered and arranged is not clear (Burton et al. 2010: Yapo 2011; Atmodjo et al. 2013). It is known that pectic polysaccharides have capacities to link to or associate with cellulose microfibrils and to matrix components such as xyloglucans as discussed above. This may indicate aspects of the synthesis of the cell wall components or relate to the organisation of cell wall matrix components that impact on cell wall architectures and properties. Several questions arise concerning cell wall matrix glycans including how diverse are the inter-linkages between glycans and what are their functional roles in impacting on overall cell wall structures and associated properties.

New molecular tools and approaches are required to reveal the developmental dynamics of these links as an aid to inform functional understanding. The study of cell wall molecular architectures is a major challenge, and studying individual cell wall components is not straightforward as extraction procedures may alter polymer structures, and all cellular contexts are likely to be lost. Efforts have been made to study cell wall components directly *in muro* using monoclonal antibodies (MAbs), and these have proven useful to indicate the diversity and dynamics of cell wall architectures. More recently, MAbs are being used to study cell wall components in glycan microarrays/glycomic approaches (Moller et al. 2007; Pedersen et al. 2012; Pattathil et al. 2012) and in chromatographic separations revealing heterogeneities and potential inter-linkages (Verhoef et al. 2008; Cornuault et al. 2014). However, in order to extend the existing libraries of MAbs and to increase probe coverage of potential oligosaccharide features found in cell walls, additional MAbs recognising novel epitopes are required.

Here, we report the isolation of a cell wall fraction from potato tubers enriched in RG-I oligosaccharides with the aim of generating MAbs. Immunisation with this fraction led to the isolation of two heteroxylan-directed MAbs. These probes, LM27 and LM28, have been used in a series of analyses, and their high sensitivity and detection capabilities allow their use to indicate that a small proportion of heteroxylan in potato tuber cell walls appears to be connected to pectic molecules and also that sub-fractions of oat spelt heteroxylan may be associated with AGPs.

## Materials and methods

### Preparation of a potato cell wall fraction enriched in RG-I oligosaccharides

Potato pulp (*Solanum tuberosum* L., obtained from Roquette, Lestrem, France) (100 g) was de-starched using an α-amylase (Termamyl 120 L, Novozyme) (10 mL at 15 U/mL) in 80 mM sodium phosphate buffer pH 6 (2 L) for 25 min at 90 °C. The suspension was cooled down to 30 °C, and the pH was brought to 4.5 by 1 M HCl. The suspension was then incubated with amyloglucosidase (*Aspergillus Niger*, A. 3042 Sigma, 1 mL, 6000 U/mL) for 17 h at 60 °C. Fresh amyloglucosidase (1 mL, 6000 U/mL) was added at 30 min and 60 min. The mixture was then cooled and the pH adjusted to 7.8 with 1 M NaOH. The de-starched potato pulp was mixed with 0.1 M NaOH (3 L) at 90 °C for 2 h. These conditions favour the β-elimination of methyl-esterified HG, resulting in the degradation of part of the HG component and enrichment in pectic RG-I. After filtration and NaOH neutralisation with 1 M HCl, the solution was concentrated to 1.5 L by rotary evaporation at 40 °C, and polysaccharides were recovered by precipitation with 70 % (v/v) ethanol overnight at 4 °C. The suspension was centrifuged, and the pellet was recovered and dissolved in water. The solution was concentrated by evaporation to 1 L to eliminate alcohol traces. The whole protocol starting with ethanol precipitation was repeated twice. Finally, the residual salts present in the solution were removed by dialysis. The salt-free solution was filtered with 3-μm membrane and freeze-dried. This fraction (1200 mg) would contain non-cellulosic polysaccharides.

The most acidic polysaccharides present in the non-cellulosic fraction were removed using diethylaminoethanol (DEAE)-Sepharose fast flow gel in batch. After pre-equilibration using 20 mM sodium acetate pH 4.5, the sample (400 mg in 40 mL H_2_O) was loaded onto the chromatography gel (150 mL). The matrix was manually stirred over 20 min to allow the most acidic polysaccharides to fix. A RG-I enriched fraction that did not bind to the gel was recovered by rinsing the gel with 4 × 100 mL of 20 mM sodium acetate buffer pH 4.5. The most acidic polysaccharides were then eluted by 4 × 100 mL of 50 mM acetate pH 4.5 + 0.6 M NaCl and discarded. The whole procedure was repeated three times and the three RG-I-enriched fractions pooled, dialysed and freeze-dried. The RG-I enriched sample (650 mg) was then treated with an endo-1,4-β-galactanase (from *Aspergillus niger*, Megazyme International, 1 mL at 2.35 U/mL) in 50 mM sodium acetate buffer pH 4.5 (130 mL) for 150 min at 40 °C, followed by an endo-arabinanase digestion (Novozyme, cloned from *Aspergillus aculeatus* in *Aspergillus oryzae*, batch PPJ4381, repurified on a Superose column using 5 mM succinate buffer pH 4, to eliminate any non-specific activity) (2.3 mL at 3.42 U/mL) in 50 mM sodium acetate pH 4.5, for 24 h at 30 °C. In both cases, the reaction was stopped by boiling the sample in order to avoid full digestion and preserve the beginning of the side chains. The sample was then dialysed for 48 h to eliminate the generated oligosaccharides and freeze-dried. This was designated as a low-branched RG-I-enriched fraction.

A sample aliquot (116 mg) was digested with a family GH28 rhamnogalacturonase (Novo Nordish Batch PPJ4478, 25 g recovery pilot plant 09/12/1993) (300 µL at 1 mg/mL) in 10 mM sodium acetate pH 4.0, for 180 min at 40 °C. The undigested polysaccharides were precipitated using 50 % EtOH at 4 °C overnight, centrifuged for 10 min at 15000 *g* and rinsed 3 times with 50 % EtOH. The EtOH-soluble fraction was concentrated by rotary evaporation at 40 °C, desalted using a column (100 × 1.6 cm) of Sephadex G-10 at 1 mL/min eluted by deionised water and freeze-dried. This fraction (designated RG-I oligosaccharides-enriched fraction or RUP (R, rhamnose; U, uronic acid; P, potato), 78 mg) was coupled to bovine serum albumin (BSA) to prepare the immunogen.

### Sugar analysis

Uronic acids were measured by the automated *m*-hydroxybiphenyl method (Thibault 1979). The difference in response of glucuronic acid (GlcA) and galacturonic acid (GalA) in the presence and absence of Na-tetraborate (11.9 g of Na-tetraborate decahydrate in 2.5 L of sulphuric acid (97–98 %) (Filisetti-Cozzi and Carpita 1991) was used to quantify them individually in purified fractions. Total neutral sugars were measured by the orcinol method with correction for the interference by uronic acids (Tollier and Robin 1979). Individual neutral sugars were analysed as their alditol acetate derivatives (Blakeney et al. 1983) by gas liquid chromatography (GLC) after hydrolysis with 2 M trifluoroacetic acid at 121 °C for 2.5 h. *Myo*-inositol was used as an internal standard.

### Carbohydrate microarrays of oligo- and polysaccharides

Carbohydrate microarrays printed on nitrocellulose were produced and quantified as described (Pedersen et al. 2012). In brief, the printed microarrays were probed with appropriate rat MAbs (10-fold dilution in phosphate-buffered saline (PBS) containing 5 % w/v milk powder (MPBS). Secondary anti-rat antibodies conjugated to alkaline phosphatase (Sigma) were diluted in MPBS to 1/5000. Developed microarrays were scanned at 2400 dpi (CanoScan 8800F), converted to TIFFs, and signals were measured using Array-Pro Analyzer 6.3, Media Cybernetics software. The mean spot signals obtained from four experiments are presented in heat maps in which colour intensity is correlated to signal. The highest signal in each dataset was set to 100, and all other values were normalised accordingly as indicated by the colour scale bar.

### Immunisation procedures and generation of rat MAbs

The RUP oligosaccharides (10 mg) were coupled to BSA in order to enhance their immunogenicity following the procedure from Lees et al. (1996). The coupling efficiency was checked by the phenol sulphuric acid assay.

Rat immunisation, preparation of hybridomas and cell cloning were performed as described (Willats et al. 1998). Two male Wistar rats were injected with 25 μg of RG-I oligosaccharides coupled to BSA in complete Freund’s adjuvant administered sub-cutaneously on day 0, with the same amount administered with incomplete Freund’s adjuvant on days 28 and 63. On day 133, the rat was given a pre-fusion boost of 1 mg immunogen in PBS by intraperitoneal injection. Spleen lymphocytes were isolated 3 days later and fused with rat myeloma cell line IR983F (Bazin 1982). Hybridoma cells were selected by enzyme-linked immunosorbent assay (ELISA) using the coupled oligosaccharides as the immobilised antigen and this led to the isolation of rat MAbs LM27 and LM28. The determination of the immunoglobulin isotypes revealed that both antibodies are IgM.

### MAbs

Established MAbs used included rat MAbs LM2 (Smallwood et al. 1996), LM11 (McCartney et al. 2005), LM19 (Verhertbruggen et al. 2009; Marcus et al. 2010) and mouse MAb INRA-RU2 (Ralet et al. 2010).

### Plant-derived oligo- and polysaccharides and materials for immunochemical assays

Various sources of plant cell wall polysaccharides were used for the characterisation of LM27 and LM28 using ELISAs. These included tamarind xyloglucan (100403, Megazyme International), maize xylan (McCartney et al. 2005), birchwood xylan (X0502, Sigma-Aldrich), rye arabinoxylan (20601b, Megazyme International) and oat spelt xylan (95590, Sigma-Aldrich). Individual xylan-derived aldouronic acid oligosaccharides were a kind gift from Sanna Koutaniemi (Koutaniemi et al. 2012), and a mixture of aldouronic acids (tri:tetra:penta—2:2:1) was obtained from Megazyme.

The *Arabidopsis thaliana* triple mutant in *gxm1gxm2gxm3* was generated by crossing single mutants prepared in Li et al. (2013). The insertion lines are SALK_087114 (gxm1, At1g33800), SALK_084669 (gxm2, At4g09990) and SALK_050883 (gxm3, At1g09610). Plants from the triple mutant line were grown for 6 weeks, and 5-cm of basal inflorescence stem was harvested. The alcohol-insoluble residue (AIR) was obtained and pre-treated with alkali and digested with a GH11 xylanase as described (Mortimer et al. 2010). After GH11 digestion, resulting sugars were derivatised by 9-aminopyrene-1, 4, 6-trisulfonate (APTS) and analysed by DNA sequencer-Assisted Saccharide analysis in high throughput, DASH (Li et al. (2013). GH11 products of stem AIR digestion were deuteropermethylated and analysed by MALDI-TOF-Mass Spectrometry as described (Tryfona et al. 2010).

### Immunochemical analyses

#### ELISAs

Enzyme-Linked Immunosorbent Assays (ELISAs) were performed as described (Cornuault et al. 2014). For isolated polysaccharides, 100 µL of polymers at the indicated concentrations in PBS (phosphate-buffered saline: 137 mM NaCl, 2.7 mM KCl, 10 mM Na_2_HPO_4_, 2 mM KH_2_PO_4_) was coated overnight at 4 °C on to microtitre plates. In some cases, enzymes (a family GH11 xylanase (Megazyme International) and a family GH115 xylan glucuronidase (kind gift of Harry Gilbert, Newcastle University)) were used to pretreat samples prior to ELISAs.

#### Sandwich ELISAs

Primary MAbs (LM2, LM11, LM27 and LM28) were coated in 1:5 dilution in PBS at 4 °C overnight, 100 µL/well. After incubation, the plates were washed thoroughly with tap water and then blocked using 200 µL/well with MPBS for 2 h at room temperature (RT). As a control of the blocking efficiency, some wells were directly blocked with MPBS without any MAb pre-coating. The plates were washed with tap water and incubated with 100 µL of 50 µg/mL solution of polysaccharides in MPBS. Another wash step was performed before the incubation with directly coupled LM28-horseradish peroxidase (HRP) antibody. The LM28-HRP antibody was diluted 1/25 in MPBS and incubated at 100 µL/well for 1 h at RT.

### LM28 antibody purification and coupling to horseradish peroxidase (HRP)

LM28 was purified using the euglobulin precipitation protocol which involved hybridoma cell culture supernatant (250 mL) being dialysed for 3 days at 4 °C with 2 mM sodium phosphate buffer pH 6.0. The precipitated IgM was then centrifuged at 4 °C, 4000*g* for 10 min. The pellet was re-suspended and rinsed twice with cold 2 mM sodium phosphate buffer pH 6.0. Finally, the pellet was re-suspended in 10 mL of 1X PBS, centrifuged at 2000*g* for 10 min at RT. The supernatant was collected and aliquoted for storage. The efficiency of the purification was checked via SDS-PAGE, and the activity of the purified antibody was checked via immuno-dot assay on oat spelt glucuronoarabinoxylan (GAX). The concentration of the purified LM28 antibody was estimated by absorbance reading at 280 nm. The coupling of LM28 to HRP was performed using the EZ-link™ Plus Activated Peroxidase kit (Thermo Scientific) following manufacturer instructions.

### Epitope detection and anion-exchange chromatographies

Epitope detection chromatography (EDC) analysis was performed as described (Cornuault et al. 2014) using a 1-mL HiTrap ANX FF column (GE Healthcare, 17-5162-01). As this analysis was performed on pre-purified samples, only 50 µg of oat spelt xylan, birchwood xylan or low-branched RG-I-enriched fraction was injected. For optimised separation, the samples were eluted at 1 mL/min using a two-step gradient starting with 20 mM sodium acetate buffer pH 4.5 from 0 to 25 min with a step change to 50 mM sodium acetate buffer pH 4.5 at 25 min with the onset of a linear gradient of 0–50 % 0.6 M NaCl to 73 min followed by a second step from 73 to 83 min of a linear gradient from 50 to 100 % 0.6 M NaCl. The salt gradient remained at its maximum (50 mM sodium acetate buffer pH 4.5, 0.6 M NaCl) from 83 to 96 min.

For comparison of EDC profiles with chemical assessment of sugar content, a 30 mL anion-exchange column (DEAE-Sepharose Fast Flow 16 × 150 mm) was used. 10 mg of low-branched RG-I-enriched fraction was injected. The sample was eluted at 1 mL/min using a two-step gradient starting with 20 mM sodium acetate buffer pH 4.5 from 0 to 110 min with a step change to 50 mM sodium acetate buffer pH 4.5 at 110 min with the onset of a linear gradient of 0 to 50 % 0.6 M NaCl to 275 min followed by a second step from 275 to 310 min of a linear gradient from 50 to 100 % 0.6 M NaCl. The salt gradient remained at its maximum (50 mM sodium acetate buffer pH 4.5, 0.6 M NaCl) from 310 to 350 min.

### Preparation of plant material and immunocytochemistry

Tobacco (*Nicotiana tabacum* L.) stem sections were obtained as described in Marcus et al. (2008). *Brachypodium distachyon* (L.) P.Beauv stem sections were obtained from the 5th internode of 50-day-old stem. The *Arabidopsis thaliana* (L.) Heynh. stem sections were obtained from the first centimetre of inflorescence stem of 1-month-old plant. They were fixed and embedded in resin as described (Lee and Knox 2014). Transverse sections of *A. thaliana,B. distachyon* and tobacco stems were incubated for 30 min with MPBS to prevent non-specific binding, and then washed for 5 min with PBS. Primary rat MAbs at 5-fold dilutions of hybridoma cell culture supernatants in MPBS were incubated on sections for 90 min at RT. Sections were then washed three times with PBS for 5 min. The secondary antibodies (anti-rat IgG-FITC (Sigma-Aldrich) at a 100-fold dilution were added in MPBS and incubated for 90 min in the dark. Sections were washed with PBS for three times for 5 min. To diminish sample auto-fluorescence, the sections were incubated with 0.1 % Toluidine Blue O (pH 5.5, 0.2 M sodium phosphate buffer) for 5 min. Following Toluidine Blue O labelling, sections were washed twice with PBS for 5 min, and then mounted in anti-fade reagent Citifluor AF1 (Agar Scientific). After mounting, slides were stored at 4 °C in darkness until use. Immunofluorescence was observed with a fluorescence microscope (Olympus BX61), and images were captured using a Hamamatsu ORCA285 camera (Hamamatsu City, Japan) using PerkinElmer Volocity software (PerKinElmer).

## Results

### Preparation of a potato RG-I oligosaccharides-enriched fraction for use in an immunogen

**Fig. 1:**
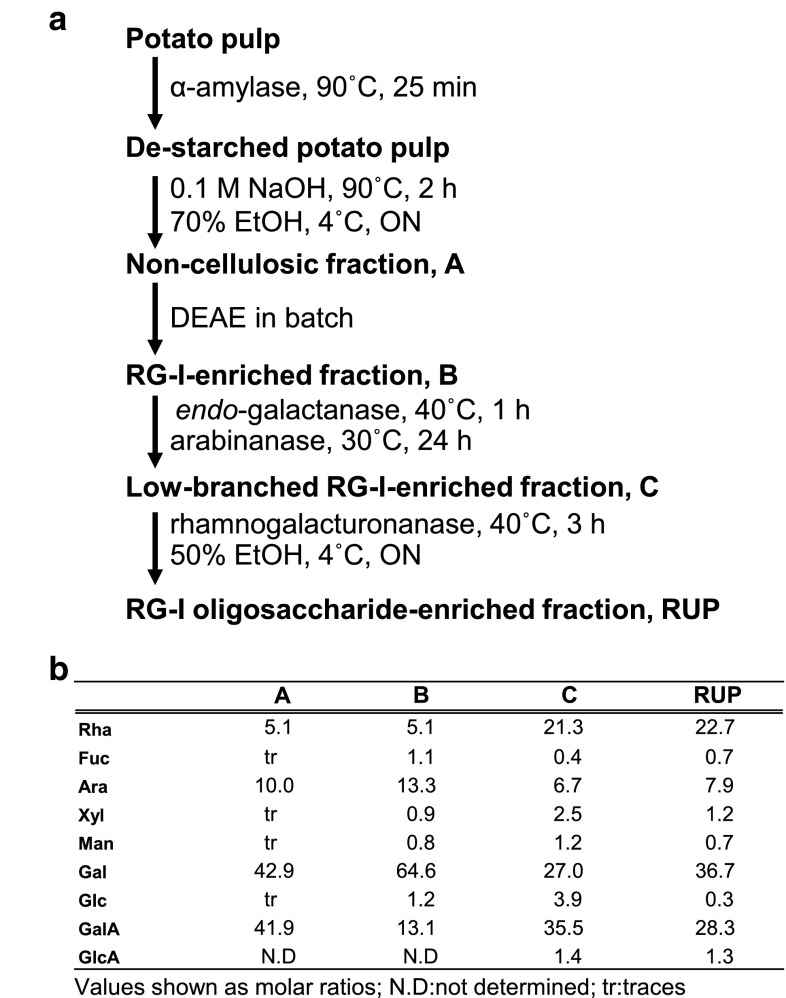
Isolation of a potato RG-I oligosaccharides-enriched fraction for preparation of an immunogen. **a** Outline of protocol followed for the production of RG-I oligosaccharides-enriched fraction. **b** Monosaccharide compositions of the non-cellulosic, RG-I-enriched, low-branched RG-I-enriched and RG-I oligosaccharides-enriched fractions. *Ara* arabinose, *Fuc* fucose, *Gal* galactose, *GalA* galacturonic acid, *Glc* glucose, *GlcA* glucuronic acid, *Man* mannose, *Rha* rhamnose, *Xyl* xylose

In order to isolate oligosaccharides from the core of RG-I structures for immunisation, a pectic fraction was extracted from potato pulp using the protocol shown in Fig. 1a. The sample was first de-starched using an α-amylase digestion followed by a treatment with alkali at 90 °C to solubilize non-cellulosic polysaccharides and to induce β-elimination reactions in highly methyl-esterified HG stretches to promote their degradation (Kiss 1974). The polymers remaining in this non-cellulosic fraction were then precipitated with 70 % EtOH and further purified using an anion-exchange batch separation to give a RG-I-enriched fraction (Fig. 1). The monosaccharide compositions of the non-cellulosic and RG-I-enriched fractions were determined (Fig. 1b). The GalA/Rha ratio that was in the region of 8.2 in the non-cellulosic fraction decreased to 2.5 in the RG-I-enriched fraction, evidencing the successful removal of homogalacturonan remnants. Both fractions were rich in galactose and, to a lesser extent, in arabinose. In order to obtain RG-I oligosaccharides containing backbone oligosaccharides, the RG-I-enriched fraction was enzymatically treated with an *endo*-galactanase followed by an *endo*-arabinanase to produce a low-branched RG-I-enriched fraction in which both galactose and arabinose contents were successfully reduced but not totally removed. The low-branched RG-I-enriched fraction was then enzymatically cleaved using a GH28 rhamnogalacturonanase generating a range of oligosaccharides of various lengths and composition. Finally, non-digested polysaccharides were precipitated using 50 % ethanol. This step was used to isolate specifically the oligosaccharides as any non-digested pectin or other non-cellulosic polymer was removed in the precipitate. The final GalA/Rha ratio of this RG-I oligosaccharides-enriched fraction was 1.25, with abundant galactose and to a lesser extent, arabinose. Xylose, fucose, glucose, mannose and glucuronic acid were also detected, each representing around 1 % of the sugar content. This fraction, designated RUP (R, rhamnose; U, uronic acid; P, potato), was coupled to BSA to prepare the immunogen.

### Isolation of xylan-directed MAbs LM27 and LM28

**Fig. 2:**
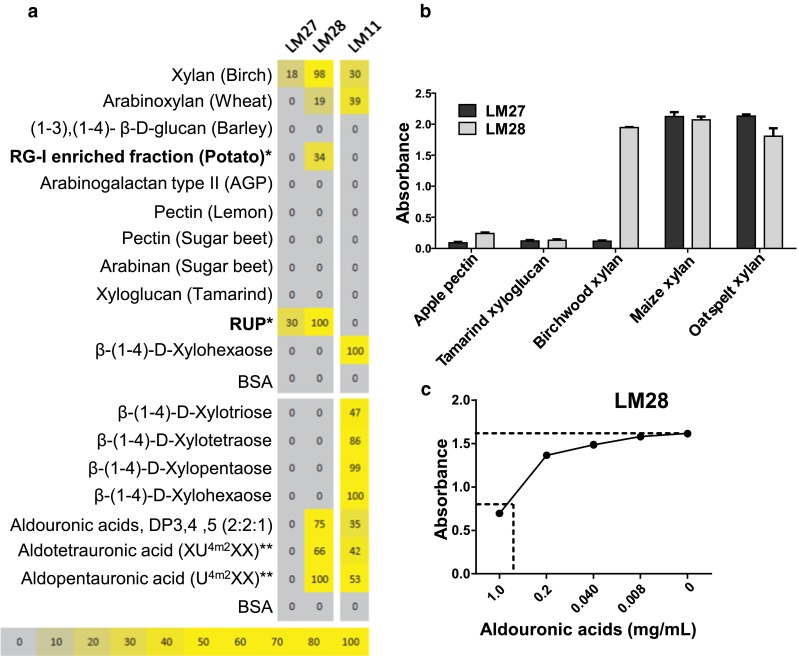
Binding of LM27 and LM28 to various cell wall glycans. **a** Carbohydrate microarray binding profile, analysing LM27, LM28 and LM11 as a control, binding to various poly- and oligosaccharides (*bold*). Mean spot signals obtained from four experiments are presented in a heatmap in which colour intensity is correlated to signal. The highest signal in each dataset was set to 100, and all other values were normalised accordingly as indicated by the colour scale bar. RG-I* is the RG-I-enriched fraction and RUP* the oligosaccharide fraction used to prepare the immunogen. *Double asterisk* indicates xylan-derived aldouronic acid oligosaccharides (Koutaniemi et al. 2012). **b** ELISA analysis of LM27 and LM28 binding to pectin, xyloglucan and an extended set of heteroxylans. Values displayed are mean of three technical replicates with SE shown as error bars. **c** Competitive inhibition ELISA of LM28 (binding to immobilied oat spelt xylan) by a mixture of aldouronic acids. Absorbance values shown are the mean of three technical replicates. The IC50 is 0.88 mg/mL

Immunisation of rats with the RUP-BSA conjugate led to the isolation of several cell lines secreting antibodies that bound to the immunogen. Two of these, designated LM27 and LM28, displayed, in addition, recognition of xylan samples and were characterised further. Analysis of binding to a number of plant polysaccharides and oligosaccharides on a glycan microarray (Pedersen et al. 2012) indicated that LM27 bound weakly to birchwood xylan. LM28 bound to birchwood xylan and the RG-I-enriched fraction from potato pulp and showed strong binding to glucuronoxylan-derived aldouronic acid oligosaccharides (XU4m2XX and U4m2XX, detailed in Koutaniemi et al. 2012) (Fig. 2a). The binding of established xylan-directed MAb LM11 (McCartney et al. 2005) is shown for comparison. To explore the recognition of heteroxylans further, LM27 and LM28 MAbs were tested with ELISA against birchwood xylan, maize kernel and oat spelt xylan/glucuronoarabinoxylan (GAX) with apple pectin and tamarind xyloglucan as controls (Fig. 2b). LM27 bound strongly and specifically to the two xylans from grass species, and LM28 bound strongly to all three heteroxylans. Competitive inhibition ELISA confirmed LM28 recognition of aldouronic acid oligosaccharides (Fig. 2c).

### LM28 binds to the glucuronosyl substitution of heteroxylan

**Fig. 3:**
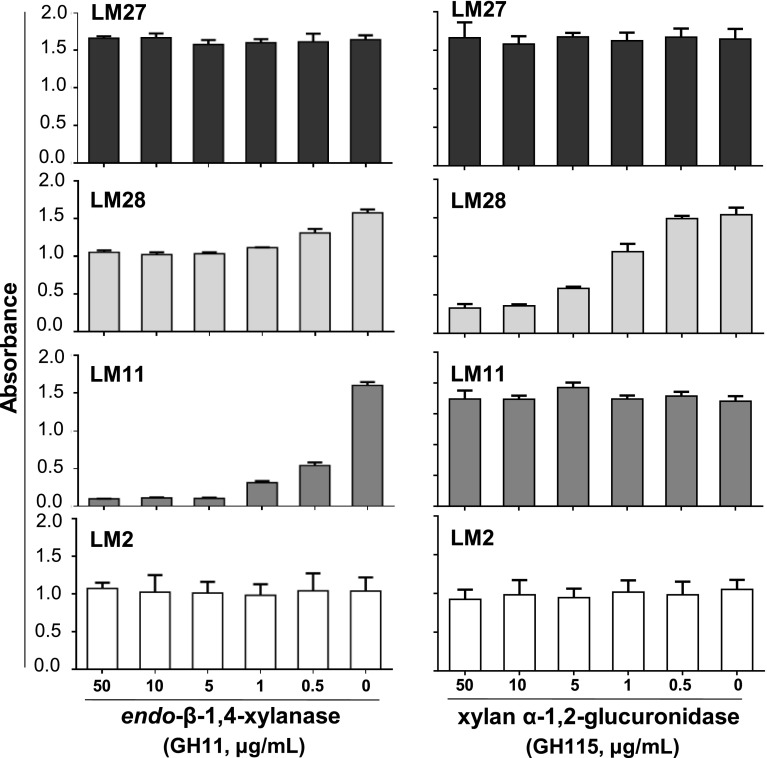
ELISA analysis of the impact of *endo*-β-1,4-xylanase (GH11) and α-1,2-glucuronidase (GH115) treatment on LM27, LM28, LM11 and LM2 (AGP) binding to oat spelt xylan. *Error bars* SE of triplicate values

**Fig. 4:**
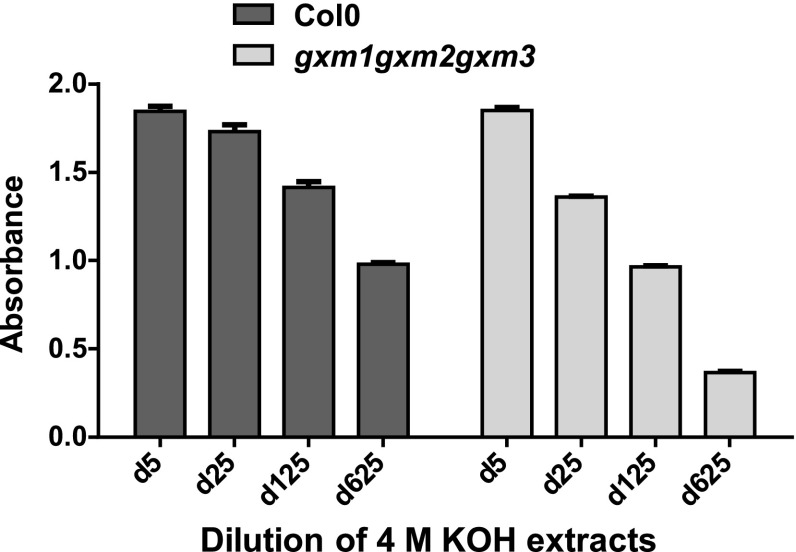
ELISA analysis of LM28 binding to 4 M KOH extracts of Arabidopsis Col0 WT and *gxm1gxm2gxm3* triple mutant. Dilutions of extracts of 1 mg AIR in 1 mL 4 M KOH were used and are indicated by d*x* (*x* being the numerical dilution factor). *Error bars* SE of triplicate values

Further analysis was carried out in order to characterise the recognition of the glucuronoxylan/GAX-related epitopes by LM27 and LM28. Oat spelt xylan was coated onto microtitre plates and treated with hydrolytic enzymes prior to ELISA analysis. The binding of xylan-directed MAb LM11 to oat spelt xylan was abolished after the digestion by an endo-β-1,4-xylanase (GH11), LM28 binding was reduced by a third, whereas LM27 binding was unaffected (Fig. 3). Pre-treatment with a family GH115 α-1,2-glucuronidase had no impact on the binding of LM11 or LM27 but led to a significant loss of LM28 binding, indicating that the LM28 epitope includes a α-1,2-GlcA substitution of xylan. The potential impact of 4-*O*-methylation of GlcA residues on xylan backbones on LM28 recognition was explored using alkali extracts from alcohol-insoluble residues of inflorescence stems of *Arabidopsis thaliana* with mutations in three glucuronoxylan methyltransferases (Urbanowicz et al. 2012; Li et al. 2013). The triple mutant *gxm1gxm2gxm3* has no detectable methylation of xylan GlcA (Fig. S1). There is only a small loss of LM28 signal in the cell wall extracts with no Me-GlcA indicating that methylation of xylan GlcA residues is not necessary for the binding of LM28 (Fig. 4).

The potential of other enzyme deconstructions of heteroxylan/GAX involving α-L-arabinofuranosidase (GH51) and endo-/exo-arabinanases (GH43) to disrupt LM27 recognition of oat xylan led to no loss of binding suggesting recognition of a structurally complex substitution of heteroxylan or the presence of an associated non-xylan structure.

### A link from RG-I to xylan: Epitope detection chromatography/sandwich-ELISA analysis of the low-branched RG-I-enriched fraction using LM28 reveals a capacity for links between xylan and pectic polysaccharides

**Fig. 5:**
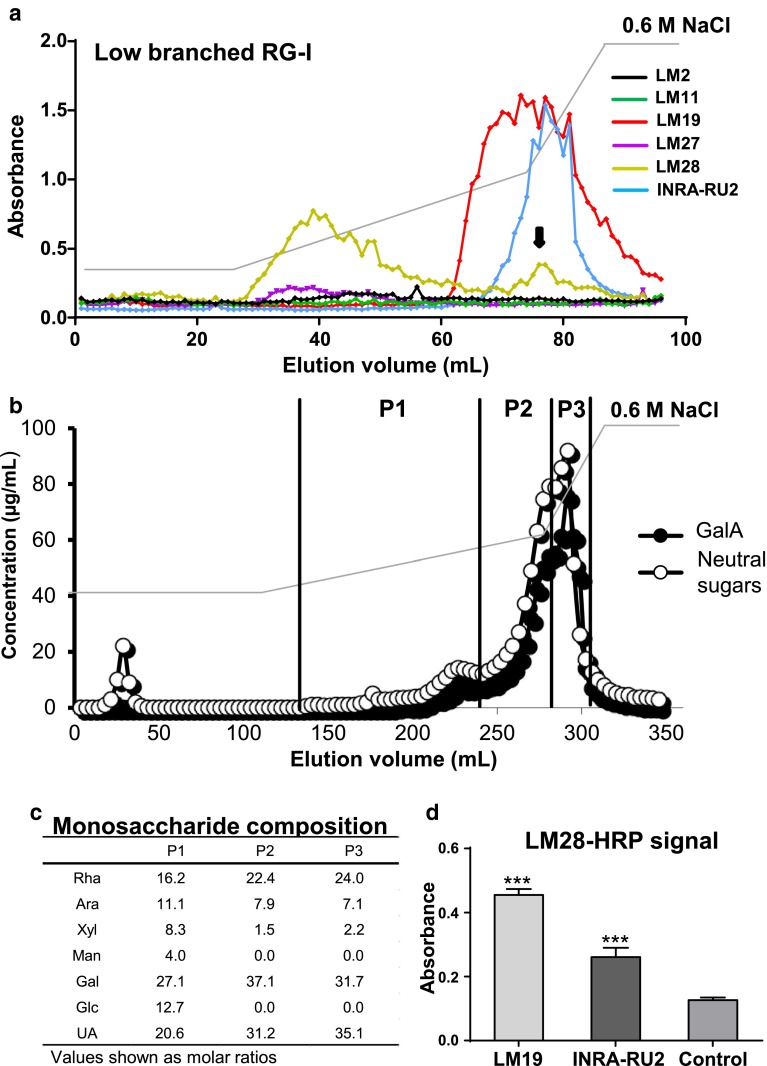
**a** EDC profile of the low-branched RG-I-enriched fraction using LM2 (AGP), LM11, LM27, LM28, INRA-RU2 (RG-I backbone) and LM19 (HG). **b** Compositional profile of the same fraction through anion-exchange chromatography determined by automated *m*-hydroxybiphenyl and orcinol methods (Thibault 1979; Tollier and Robin 1979). **c** Monosaccharide analysis of pools 1, 2 and 3 shown as P1, P2 and P3 in **b**. **d** Sandwich-ELISA of the low-branched RG-I-enriched fraction using directly coupled LM28-HRP antibodies. LM19 and INRA-RU2 were used for the capture of glycan. The control was the absence of a capture antibody. *t* test analysis of values relative to the control, (***P value <0.0005). *Ara* arabinose, *Gal* galactose, *Glc* glucose, *Man* mannose, *Rha* rhamnose, *UA* uronic acid, *Xyl* xylose

Epitope detection chromatography (EDC) is a technique that combines chromatographic separation of polysaccharides with detection of glycan epitopes and can indicate potential links between epitope-carrying polymers (Cornuault et al., 2014). Analysis of the low-branched RG-I-enriched fraction using this technique indicated the existence of two broad populations of eluted polymers as revealed by the LM27 and LM28 MAbs and previously characterised probes of pectic supramolecule domains. One moderately acidic peak eluting between 28 and 55 ml contained the LM28 and to a lesser extent the LM27 epitope, and a later eluting peak from 60 to 90 mL was identified by the LM19 (homogalacturonan) epitope, and within this from 70 to 85 mL, the INRA-RU2 (RG-I backbone)—both indicative of pectic molecules—as shown in Fig. 5a. A second, smaller peak of LM28 binding was co-incident with the pectic peak as identified by INRA-RU2 suggesting that a small component of the glucuronoxylan in the low-branched RG-I sample is associated with pectic supramolecules. To further study the occurrence of xylan in the low-branched RG-I preparation, a comparable chromatography experiment was carried out using a larger 10 ml anion-exchange column to allow the collection of more product which was then analysed by conventional methods for sugar quantitation. In this case, 10 mg of the low-branched RG-I fraction was loaded on the column, and 96 fractions of 3.6 ml were collected. Neutral sugars and GalA were determined (Fig. 5b) by automated *m*-hydroxybiphenyl and orcinol methods (Thibault 1979; Tollier and Robin 1979). The sugar composition profile did not fully match the antibody EDC profiles. These differences can be explained by the high sensitivity of various MAbs specific to a range of oligosaccharide structures compared to the chemical assays used to determine the sugar content of the fractions. The compositional profile identified three principal peaks. The first peak containing neutral sugars appears from fraction 20 to fraction 30 ml, a second peak (of GalA and neutral sugars) was not well defined and extended from fraction 200 to fraction 240 mL. The major peak (GalA and neutral sugars) was observed from 260 to 310 mL. The monosaccharide compositions of three sets of pooled fractions from 140 to 310 mL, P1, P2 and P3 (see Fig. 5b), were determined (Fig. 5c).

The P1 pool is equivalent to the region of the EDC profile that contains most of the LM28 signal and this had the highest level of xylose (Fig. 5c). Together these observations suggest that the low-branched RG-I-enriched fraction isolated from potato tubers contains small amounts of glucuronoxylan, which can be bound by LM28. The potential link between pectin and glucuronoxylan indicated by the co-elution profiles of the LM28 and INRA-RU2 epitopes (Fig. 5a) was explored further. A sandwich-ELISA using LM19 and INRA-RU2 antibodies immobilised to microtitre plates as bait was used. Incubation of these microtitre plate wells with the low-branched RG-I-enriched fraction and subsequent probing with LM28 directly coupled to horseradish peroxidase (HRP) confirmed that some LM28 epitope was present in a complex with pectin (Fig. 5d).

The accumulated observations above indicate that the fraction enriched in RG-I oligosaccharides, used as the immunogen, contained low levels of heteroxylans that led to the isolation of the LM27 and LM28 MAbs and that some of these heteroxylan structures are associated with/linked to pectic polysaccharides.

### A link to from xylan to AGPs in oat spelt xylan

To explore the potential for the occurrence of links between sub-populations of heteroxylan and other glycans further, commercial preparations of oat spelt and birchwood xylan were screened for the presence of pectic and also AGP epitopes using ELISAs. The LM2 AGP epitope was abundantly detected in oat spelt xylan (with an antibody signal equivalent to that for LM11, LM28 and LM27) but was absent from birchwood xylan (Fig. S2). LM2 binds to β-linked GlcA in AGPs (Yates et al. 1996). Pre-treatment of oat spelt xylan with α-1,2-glucuronidase resulted in no loss of LM2 signal when binding to this preparation confirming that the LM2 antibody was not binding to GlcA residues in xylan (Fig. 3).

**Fig. 6:**
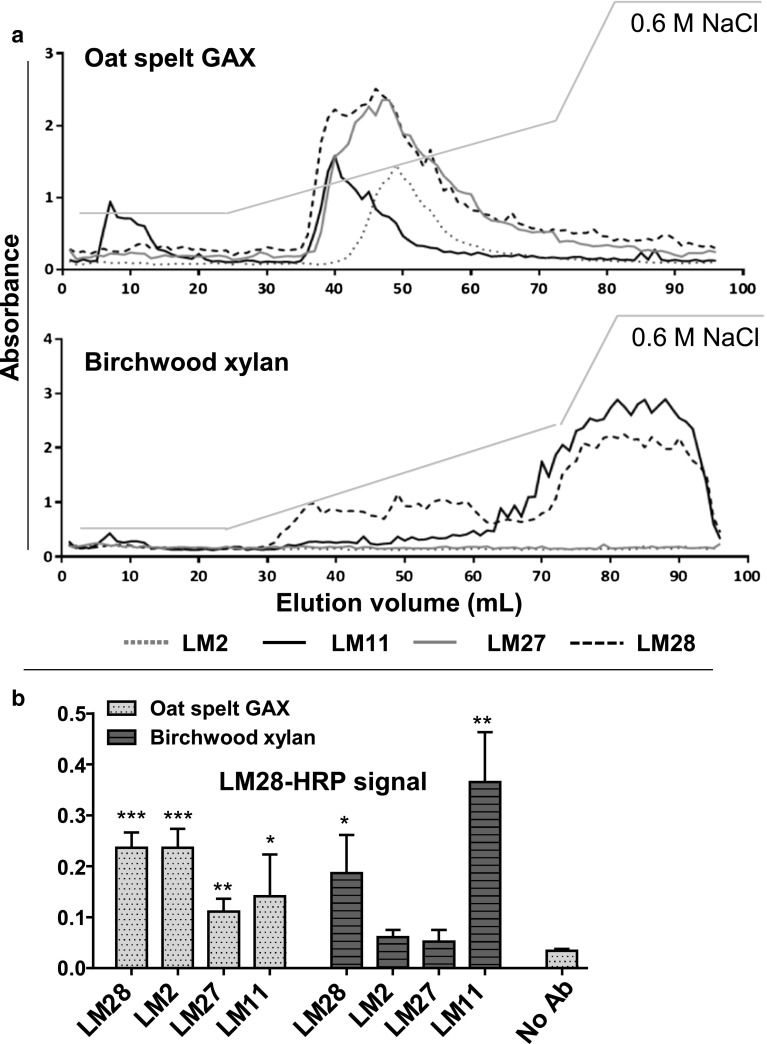
Characterisation of two commercial xylans, birchwood xylan and oat spelt GAX using antibody-based approaches. **a** Epitope detection chromatography (EDC) profiles obtained with 20 μg of oat spelt GAX and birchwood xylan using LM2, LM11, LM27 and LM28. Salt elution gradient shown as *grey line*. **b** Sandwich-ELISA analysis of oat spelt GAX and birchwood xylan using LM2, LM28, LM27 and LM11 as capturing antibodies, and captured polysaccharides were detected using directly coupled LM28-HRP antibody. No Ab is oat spelt signal in the absence of a capturing antibody. *t* test analysis of values relative to control: ***P values <0.0005, **<0.005, *<0.05

To study the potential sub-populations within xylan samples and potential linkages between heteroxylan epitopes and AGPs, EDC profiles of oat spelt xylan and birchwood xylan were generated using the mAbs LM27 and LM28 along with LM11 xylan and LM2 AGP probes (Fig. 6a). In EDC analysis of oat spelt xylan, LM11 bound to a first subset of neutral xylan. Further in the elution gradient, a more acidic pool of polymers containing the LM27, LM28, LM11 and LM2 epitopes was eluted. The acidic peak of LM11 tailed off quickly in contrast to the LM28 and LM27 peaks which were highly similar in profile. A peak of LM2 AGP detection was observed later but co-chromatographing with the large peaks of LM27 and LM28 epitopes. AGPs are acidic and may be co-eluting with acidic xylans with no connection or this may indicate the interconnection of the xylan and AGP epitopes. Potential links between acidic glucuronoxylan/GAX and AGPs were explored using sandwich ELISAs (Fig. 6b). Purified MAbs LM2, LM11, LM27 and LM28 were used to coat microtitre plate wells, incubated with oat spelt xylan and then probed with LM28-HRP. The results showed that polysaccharides captured using immobilised LM2 were detectable using LM28-HRP, suggesting that some LM28 xylan epitope is connected to the LM2 AGP epitope. A lower ELISA signal was obtained for polymers captured using LM11 and LM27 but these were above background levels. These observations suggest that the large peak defined by the LM28 epitope may contain a spectrum of molecules with early eluting glycans rich in the non-acidic LM11 epitope, and late eluting glycans may contain some attached AGP molecules.

Comparative experiments (EDC and sandwich ELISAs) carried out on birchwood xylan led to a distinct set of observations (Fig. 6). The LM27 and LM2 AGP epitopes were not present confirming earlier observations. The LM11 epitope was associated with a large peak, eluting later (more acidic) than oat spelt xylan. The LM28 glucuronoxylan epitope was detected in this late eluting peak co-incident with LM11 but also in an early eluting peak in a similar position to its elution with the sample of oat spelt xylan. These EDC profiles indicate the presence of two separate glucuronoxylans in the birchwood samples with the later eluting polymer presumably having a higher density of GlcA residues.

### *In situ* detection of the LM27 and LM28 epitopes

**Fig. 7:**
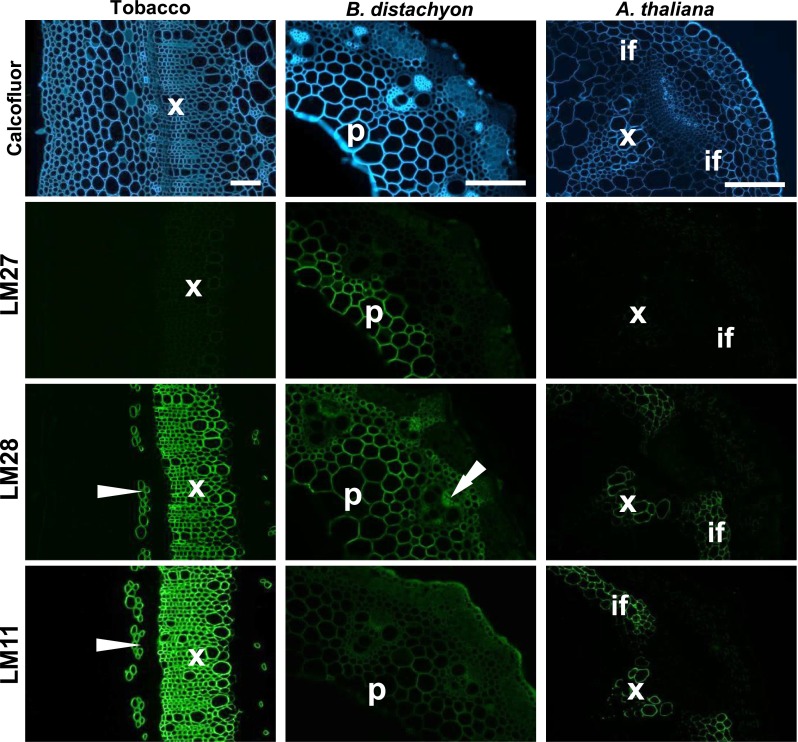
Immunodetection of the LM27 and LM28 heteroxylan epitopes in plant materials. *Top row* shows Cacofluor White fluorescence micrographs of transverse sections of tobacco stem, *Brachypodium distachyon* stem and *Arabidopsis thaliana* inflorescence stem*. Bars* 100 µm. *Second row* shows indirect immunofluorescence labelling with LM27 of equivalent sections with the epitope only being detected in cell walls of inner parenchyma cells (p) of *B. distachyon. Third row* shows indirect immunofluorescence labelling by LM28 of equivalent sections with the epitope detected in secondary cells walls of phloem fibres (*arrow head*) and xylem cells (x) of tobacco stem, a wide region of parenchyma cell walls and phloem cells (*double arrowhead*) in *B. distachyon* stem and secondary cell walls of xylem and interfascicular fibres (if) of *A. thaliana* stems. Bottom row shows equivalent immunofluorescence detection of the established heteroxylan LM11 epitope in the stems of these three species

Immunolabelling of plant materials with LM27 and LM28 was carried out to study the potential locations and functions of these heteroxylan epitopes *in muro*. Analysis of tobacco and Arabidopsis stem sections using LM28 revealed recognition of secondary cell walls of xylem and fibre cells consistent with the recognition of xylan and similar to the LM11 xylan epitope (Fig. 7). In these sections of dicotyledon stems, no binding was observed for LM27. In an example of grass species with GAX in primary cell walls, LM27 and LM28 bound to different regions of the parenchyma in transverse sections of *Brachypodium distachyon* stems. The LM28 epitope was detected abundantly in primary cell walls of the inner parenchyma region and also cell walls of parenchyma surrounding vascular bundles and also groups of fibre cells between vascular bundles and the epidermis. In contrast, the LM27 epitope was restricted to the cell walls of the inner parenchyma region alone (Fig. 7). In the stem of *B. distachyon*, the LM27 and LM28 MAbs bound more strongly than LM11, the epitope of which was most abundant in the outer epidermal cell walls.

## Discussion

### The pursuit of MAbs to plant cell wall glycans

The method of choice for the production of MAbs to carbohydrate structures requires the preparation of a neoglycoprotein immunogen using a defined oligosaccharide. However, producing sufficient quantities of structurally defined oligosaccharides by isolation or chemical synthesis routes is a challenging task, and the isolation of mixtures of oligosaccharides from complex biological materials remains a valuable way forward. The approach described here involved the isolation of a fraction enriched in RG-I oligosaccharides with the aim of isolating MAbs to novel RG-I epitopes covering the junction of side chains with the rhamnogalacturonan backbone. It is therefore of interest that one of the isolated MAbs is directed to an epitope of heteroxylan and one is associated with heteroxylan. This is not too surprising as the pool of oligosaccharides that was isolated for immunogen preparation was complex and contained xylose, mannose, fucose and glucuronic acid which are not found in RG-I and is therefore likely to have contained, at low abundance, fragments of heteroxylan, and these polymers may be immunodominant. LM28 is a glucuronoxylan-directed MAb as confirmed by glycan microarray, hapten inhibition and enzyme deconstruction analyses. Regarding the specificity of the LM27 MAb, its epitope is most abundant in grass cell wall xylan preparations/GAXs, and analyses indicate the recognition of a yet unknown side chain substitution of GAX or an associated macromolecule. *Invitro*, no enzyme treatments were found to alter LM27 binding to oat xylan, indicating that the recognised epitope is possibly a structurally complex GAX structure. As the oligosaccharides used for immunisation were generated using enzyme treatments, their final structure may be different from native RG-I molecules. The procedures used for oligosaccharide isolation may also have increased the relative abundance of uncommon structures that were resistant to enzymatic treatment and rare in the original sample. The discovery that the LM2 AGP epitope is abundant in oat spelt xylan suggests the possibility that the LM27 epitope is an AGP-like epitope in xylan preparations, although LM27 does not bind to the sample of type II arabinogalactan (gum Arabic) that is included on the microarray (Fig. 2a).

### Functional significance of inter-linkages between cell wall matrix glycans

The LM28 MAb has allowed a potential link between glucuronoxylan and pectic supramolecules to be identified in potato tuber cell walls. It is also possible that a sub-fraction of the pectic glycans in the potato RG-I preparation is also attached to the LM27 epitope—but that this is below the level of detection using the current approaches. The sandwich-ELISA analysis of an oat spelt xylan preparation suggested that both the LM27 and LM28 epitopes may be linked to AGP molecules.

Xylans are abundant in secondary cell walls of dicotyledonous plants and both primary and secondary cell walls of grasses where heteroxylan is present in the form of GAX. The specific roles of heteroxylan in secondary cell walls are proposed to be cross-linking of cellulose microfibrils to make tough composites that withstand compressive forces. In primary cell wall matrices, functions are far from clear, and heteroxylan or GAX may take on some of the roles in cell wall matrices that are carried out by pectic molecules in dicotyledons. These may relate to the construction of cellulose microfibrils and cell wall assembly, allowing cell expansion, controlling other aspects of matrix properties and cell adhesion. Although not abundant in primary cell walls of dicotyledons, low levels of xylans do occur in primary cell walls (Hervé et al. 2009) and possibly have specific functions.

Although there are numerous reports of pectin links to xyloglucan, a structural linkage of xylan to other matrix molecules has only recently been identified (Tan et al. 2013), and relevant enzymatic activities have been proposed (Franková and Fry 2013). The Arabinoxylan pectin arabinogalactan protein 1 (APAP1) of Arabidopsis cells is a very low-abundance molecule, and the links here observed in potato tuber cell walls involving xylan are also likely to be of low abundance and hence not previously documented. The low abundance of links between subsets of pectic supramolecules attached to subsets of heteroxylan may reflect a remnant of an aspect of the biosynthesis of cell wall molecules or cell wall assembly, an aspect of cell wall architecture that is required at a few locations or a small element of signalling systems that conveys information on the status of the cell wall matrix. Defined MAbs and their use in methods such as EDC will be useful tools to explore these factors further.

## Conclusion

Two new MAbs have been isolated. LM27 binds to grass GAX samples and its epitope is proposed to be a complex substitution of heteroxylan or is an epitope carried by as yet unknown attached molecule. LM28 binds to a glucuronosyl-containing epitope of heteroxylan. These probes bind with high avidity to their respective epitopes and are complementary to previously characterised heteroxylan-directed antibodies LM10 and LM11 (McCartney et al. 2005) and INRA-AX1 (Guillon et al. 2004) that bind to the backbone of xylans and also INRA-UX1 that requires alkali treatment for recognition of glucuronoxylan in plant cell walls in situ (Koutaniemi et al. 2012). Using EDC and sandwich-ELISA approaches, we have used LM27 and LM28 to demonstrate the potential attachment of a sub-fraction of potato tuber heteroxylan to pectic supramolecules and a sub-fraction of oat spelt xylan to AGP. LM27 and LM28 are therefore both useful molecular tools to study the significance and developmental dynamics of interlinks between heteroxylans and other cell wall matrix glycan classes.

### *Author contribution statement*

VC, FB, MCR and JPK conceived and designed research. VC, FB, MGR, SEM, TAT, JX, MJC and NFB conducted experiments. VC, MGR, WGTW, PD, MCR and JPK analysed data. VC, MCR and JPK wrote the manuscript. All authors read and approved the manuscript.

## Electronic supplementary material

Supplementary material 1 (PDF 120 kb)

## Abbreviations

AGP: Arabinogalactan-protein
EDC: Epitope detection chromatography
ELISA: Enzyme-linked immunosorbent assay
GalA: Galacturonic acid
GAX: Glucuronoarabinoxylan
GlcA: Glucuronic acid
GH: Glycosyl hydrolase
HG: Homogalacturonan
HRP: Horseradish peroxidase
MAbs: Monoclonal antibodies
PBS: Phosphate-buffered saline
RG-I: Rhamnogalacturonan-I
