# Supplementary material for: Monoclonal antibodies indicate low-abundance links between heteroxylan and other glycans of plant cell walls
Source: Planta. 2015 Jul 25;242(6):1321–34. doi: 10.1007/s00425-015-2375-4 (PMC4605975; doi:10.1007/s00425-015-2375-4)
Supplement: Supplementary file 1 — Supplementary material 1 (PDF 120 kb) [file 425_2015_2375_MOESM1_ESM.pdf]

Electronic supplementary material for Cornuault *et al*. Monoclonal antibodies indicate low-abundance links between heteroxylan and other glycans of plant cell walls. Planta

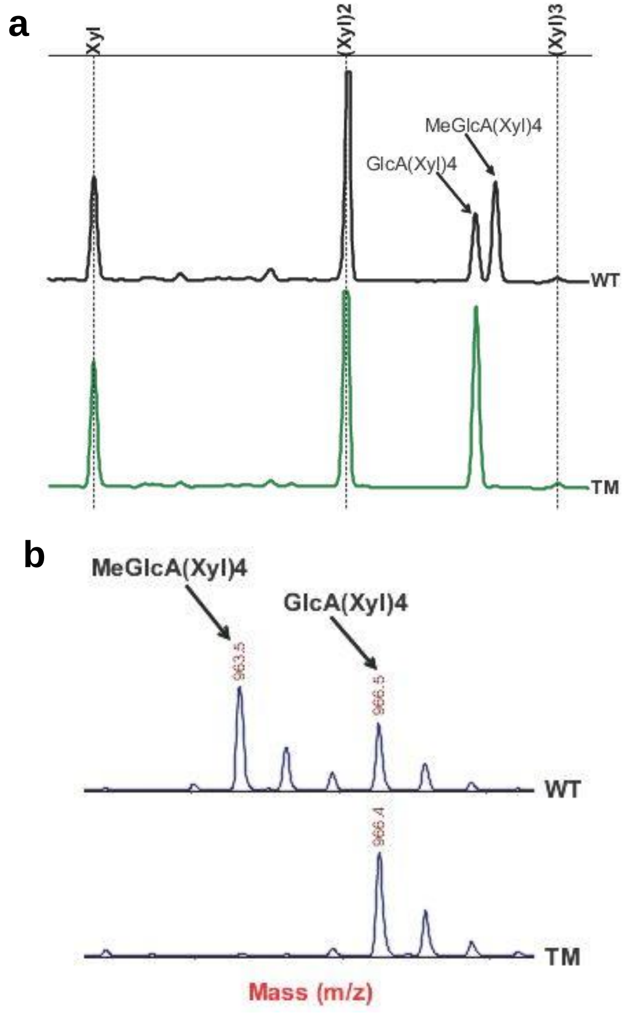

Fig. S1 The Arabidopsis DUF579 triple mutant (TM) *gxm1gxm2gxm3* has no detectable methylation of GlcA in xylan extracted from stems. A. DASH capillary electrophoresis of GH11 digest of Arabidopsis stem xylan from wild type or triple mutant plants. B. MALDI-ToF Mass spectrometry of deuteropermethylated GH11 digest of Arabidopsis stem xylan, showing the absence of MeGlcA decorations on xylan in the TM. Wild type (WT), triple mutant (TM)

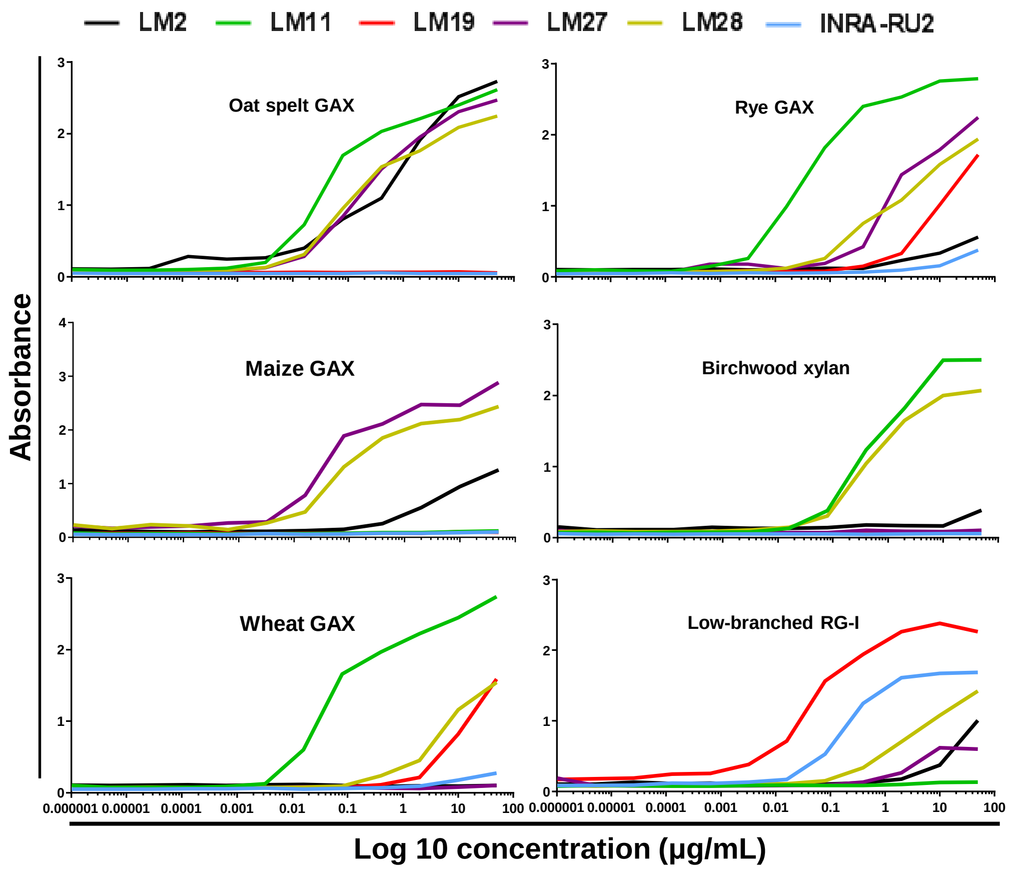

Fig. S2 ELISA analysis of the LM2 AGP, LM11, LM27, LM28 xylan, LM19 HG and INRA-RU2 RG-I epitopes in a range of isolated xylan preparations and comparison with the low-branched potato RG-I preparation. Polysaccharides coated onto plates with 5-fold dilutions from 50 µg/mL
